# Spatial–temporal evolution, drivers, and pathways of the synergistic effects of digital transformation on pollution and carbon reduction in heavily polluting enterprises

**DOI:** 10.1038/s41598-025-96834-6

**Published:** 2025-04-08

**Authors:** Wei Mai, Lixin Xiong, Ban Liu, Shengqi Liu

**Affiliations:** 1 Business School, Central South University of Forestry and Technology, Changsha, 410004 China; 2 Business School, Bangor University, Bangor, LL57 2DG UK

**Keywords:** Yangtze River Economic Belt (YREB), Coordinated pollution and carbon reduction, Enterprise digital transformation (EDT), Environmental economics, Sustainability

## Abstract

Under the “dual carbon” goals, heavily polluting enterprises face dual pressures to reduce both pollution and carbon emissions, necessitating the urgent exploration of effective pathways for coordinated emission reductions. This study investigates the potential of digital transformation in enterprises to achieve synergistic emission reductions. First, the entropy method is employed to measure enterprise digitalization and pollutant levels, and the spatial–temporal evolution characteristics of regional coordinated emission reductions are analyzed. Subsequently, using panel data from heavily polluting enterprises in the Yangtze River Economic Belt, the study examines the impact of digital transformation on pollution and carbon reduction, its underlying mechanisms, and the moderating effects of environmental policies on these relationships. Robustness tests confirm the synergy between carbon and pollution emissions. The findings reveal that digital transformation contributes to the synergistic reduction of carbon and pollutant emissions in enterprises, primarily through two pathways: the coordinated integration of internal innovation resources and the collaborative engagement in external innovation networks. Furthermore, air pollution control policies and low-carbon city initiatives significantly enhance the synergistic emission reduction effects of digitalization. Interestingly, heavily polluting enterprises located in the downstream regions of the Yangtze River, those with smaller operational scales, or those facing strong financing constraints, demonstrate more pronounced synergistic emission reduction effects through digital transformation. Based on these conclusions, we recommend that governments focus on strengthening either “pollution reduction” or “carbon reduction” policies, as either alone can yield dual emission reduction benefits. Additionally, tailoring regional emission reduction policies to local conditions can maximize economic and environmental benefits.

## Introduction

Global climate change and environmental degradation stand as critical challenges in the contemporary world, particularly for developing countries. Striking a balance between economic growth and stringent ecological governance has become a key issue demanding urgent solutions. The dual goal of reducing both pollutants and carbon emissions presents substantial practical difficulties, underscoring the necessity of exploring effective synergistic emission reduction pathways to advance sustainable development. In recent years, digital transformation has increasingly been regarded as a viable solution to these challenges. By incorporating advanced digital technologies, many nations and enterprises have achieved remarkable progress in enhancing resource efficiency and reducing emissions^1^. These efforts have not only significantly curtailed greenhouse gas and carbon emissions but have also made substantial contributions to achieving environmental sustainability objectives.

**Fig. 1:**
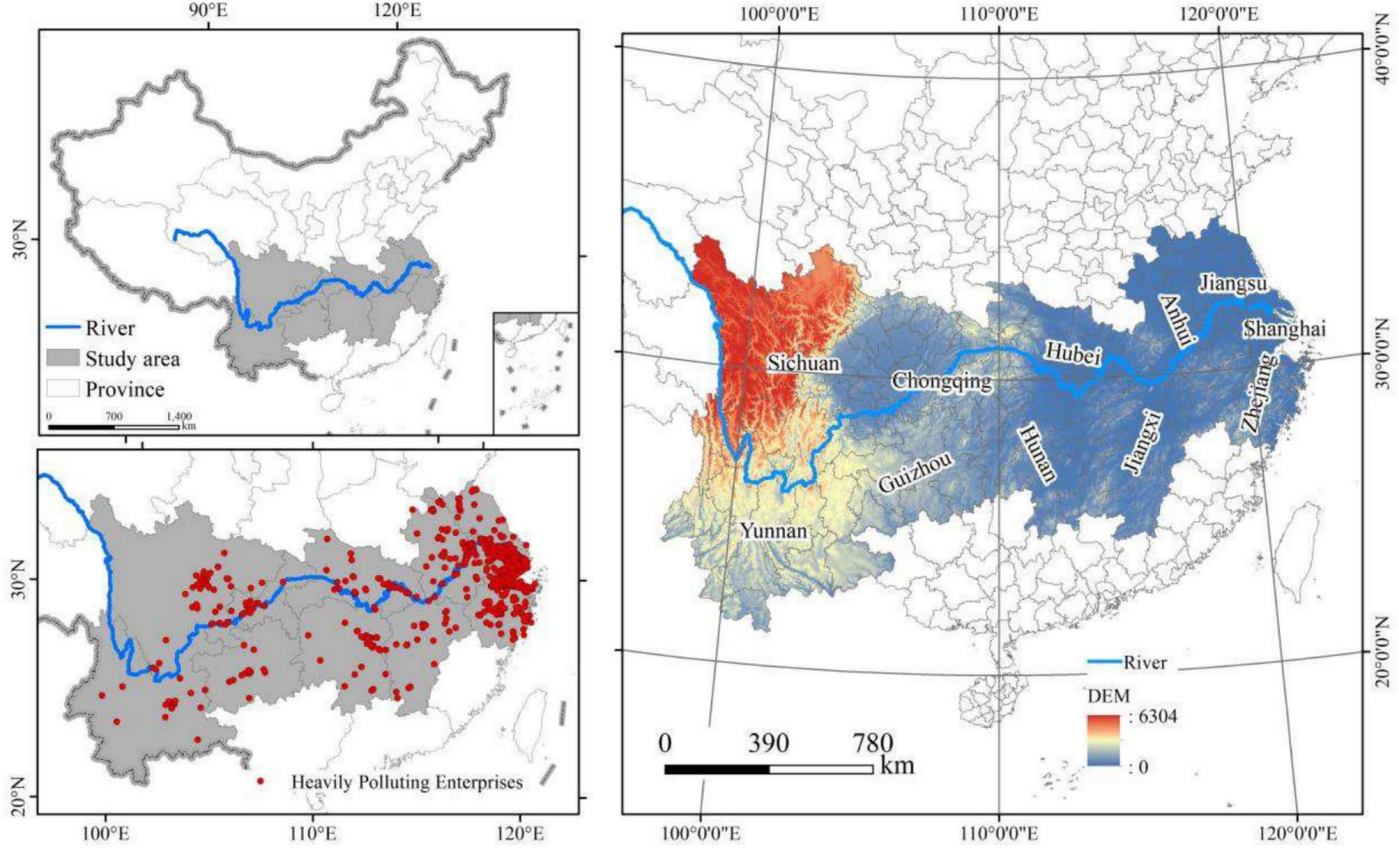
The Yangtze River Economic Belt and its major polluting enterprise locations. Software version: ArcMap 10.8, URL: https://www.esri.com/zh-cn/arcgis/products/index This map is produced based on the standard map with the review number GS(2024)0650, downloaded from the National Geographic Information Public Service Platform (website: https://www.tianditu.gov.cn/), and the base map has not been modified.

The Yangtze River Economic Belt (YREB) is a densely integrated economic corridor that spans 21.4% of China’s total land area, supporting 43.1% of the nation’s population and contributing 46.5% of its economic activities^2^. This region also serves as a major concentration of heavily polluting enterprises, facing dual pressures to manage both pollutant and carbon emissions^3^. To move away from the traditional development model characterized by high resource consumption and high pollution, many heavily polluting enterprises in the region have been compelled to adopt digital transformation strategies. Figure 1 illustrates the geographical location of the YREB within China, along with the density of heavily polluting enterprises across the region. The YREB encompasses both the economically developed and strategically advantageous Yangtze River Delta, as well as less developed mountainous areas. By selecting the YREB as the study area, this research provides valuable insights and references for other developing countries seeking sustainable growth strategies^4^.

While existing research has examined the role of digital transformation in environmental governance, several research gaps remain. First, most studies have focused on the relationship between digital transformation and single carbon emissions, with relatively little attention given to its impact on the synergistic reduction of both carbon and pollutant emissions. Second, there is a lack of holistic perspectives on the coordination of pollution and carbon emission reduction at the enterprise level^5^, and the underlying mechanisms through which digital transformation facilitates such coordination remain unexplored. This study, based on data from heavily polluting enterprises in the Yangtze River Economic Belt (YREB), focuses on the role of digital transformation in achieving synergistic emission reductions. It aims to address the following key questions:

1. Can corporate digital transformation promote the synergistic reduction of carbon and pollutant emissions?
2. Through which mechanisms does digital transformation influence synergistic emission reduction behavior?
3. What role does government policy play in this process? Are there significant differences in outcomes among enterprises with varying characteristics?

By addressing these questions, the study seeks to provide an objective understanding of the environmental benefits that digital transformation can bring in the global context. The findings will offer practical insights for enterprises in China and other emerging economies to enhance their participation in environmental initiatives.

## Literature review

### Digital transformation

Digital transformation is characterized by the application of digital technologies and the use of data elements. Scholars argue that the evolution of digital transformation can be divided into three stages: informatization, digitization, and digital transformation^6,7^. These terms differ in terms of time, concepts, and domains ^8,9^. At the micro-level, research primarily focuses on three aspects of enterprise digital transformation: driving factors, implementation pathways, and performance effects. At the micro-level, research primarily focuses on three aspects of enterprise digital transformation: driving factors^10^, implementation pathways^11^, and performance effects^12^. Regarding its impacts, scholars have concentrated on the specific effects of digital transformation on total factor productivity^13^, resource allocation efficiency^14^, and human capital efficiency within enterprises^15^. These studies indicate that digital transformation can effectively enhance resource utilization and production efficiency through technological advancements and process optimization, thereby bringing significant performance improvements to enterprises. In terms of driving factors, it is widely acknowledged in academic circles that digital transformation, as a vital development strategy, not only improves enterprise performance but also significantly reshapes the production efficiency and competitive landscape of manufacturing firms. Cost reduction and efficiency improvement have become core objectives for modern enterprises^16^. While driving technological innovation and industrial structural adjustments, digital transformation also provides new growth momentum for firms amidst fierce market competition^17^.

Accurately quantifying the degree of enterprise digital transformation has become a research hotspot in academia. Existing studies primarily employ methods such as survey-based approaches^18^, dummy variables^19^, single-index methods^20^, digital maturity models^21^, and intangible asset ratio approaches to measure the extent of enterprise digital transformation^22^. In recent studies, the application of text analysis methods has become increasingly prevalent. These approaches often involve analyzing the frequency of mentions of core foundational technologies related to digital transformation—artificial intelligence, blockchain, cloud computing, and big data (collectively referred to as “ABCD” technologies)—in corporate annual reports to gauge trends in enterprise digital transformation^23–25^. Moreover, researchers have employed advanced techniques such as machine learning and large language models to further enhance these measurements^26^. Although digital transformation is a prominent research focus, the measurement of enterprise digital transformation is still in its preliminary exploratory phase. Various measurement methods have their respective advantages, with text analysis being particularly widely used. However, no authoritative measurement framework has yet been established, nor have widely recognized measurement results been produced. The primary challenge lies not in the design of measurement frameworks but in the availability of reliable foundational data sources.

### Synergy in the reduction of pollution and carbon emissions

In most studies, the governance of pollution in enterprises referred to as “pollution reduction”^27^, and the control of greenhouse gas emissions, referred to as “carbon emission reduction”^28^, are often treated as two independent and distinct issues. However, the common causes of air pollution and carbon emissions have led to an increasing focus on the research of synergy reduction^29^. Some studies have emphasized innovative measurements by using methods such as CGE models^30^, LDMI models^31^, two-dimensional quadrant diagrams^32^, and SBM models to assess the synergy index^33^, as well as to evaluate the synergistic effects^34^. Explore the impact of environmental tax policy pilots^35^, carbon emission trading policy pilots^36^, ecological regulations^37^, and digital economy on the synergistic effects at the regional level^38^. However, research on advancing the synergy progress of carbon emission and pollution reduction is still relatively scarce^39^.

### Synergy in the reduction of pollution and carbon emission driven in enterprise digital transformation

Focusing on the relationship between digital transformation and pollutant emissions, substantial research at the macro level demonstrates that digital transformation significantly promotes carbon reduction and facilitates green development through mechanisms such as energy substitution effects^40^, technological effects^41^, and economies of scale^42^. However, at the micro level, studies on how digital transformation achieves the synergies between pollution reduction and carbon reduction are relatively sparse. A limited number of papers have examined the impacts of government subsidies^43^, environmental regulations^44^, and technological innovation on synergistic emission reduction^45^. Existing studies suggest that green technological innovation, resource allocation efficiency, and environmental information disclosure are key mechanisms by which digital transformation reduces pollutant emissions^46,47^. Additionally, the role of regional synergies in enabling emission reduction through digital transformation is particularly prominent. Some scenario-based simulation studies highlight the critical influence of environmental-economic policies in realizing these synergistic effects^43^. From the perspective of enterprise clustering, research has shown that when the industrial concentration of heavy metal polluting enterprises exceeds a certain threshold, digital transformation can significantly drive these firms to reduce pollutant emissions^48^. Currently, the scarcity of micro-level data has resulted in a limited body of literature on the measurement of synergistic relationships. A standardized framework for measuring and validating the effects of coordinated emission reductions has yet to be established.

### Research gaps and innovations

While existing studies have investigated the relationship between digitalization and pollutant emissions at regional or industry levels, a significant research gap remains regarding the digitalization of enterprises at the micro level and its specific impact on pollutant emissions. Even studies that address the effects of enterprise digitalization on carbon reduction predominantly use methods that infer enterprise-level digitalization indicators by allocating industry-level digitalization inputs based on predetermined weights^49^. However, this approach fails to capture the actual digitalization levels of enterprises directly, thus limiting its accuracy and relevance. Furthermore, most research on the environmental impacts of enterprise digitalization has focused exclusively on carbon emissions, neglecting the potential synergistic effects between carbon emissions and other types of pollutant emissions. The marginal contributions of this study are as follows to address these research gaps.

Firstly, this paper aligns with the growing wave of digital transformation, focusing on the micro-level perspective of enterprises to systematically explore the relationship between enterprise digital transformation and synergistic pollution reduction, thereby enriching and extending the body of research on the environmental effects of digital transformation in enterprises. Secondly, using the Inverse Distance Weighted (IDW) method in ArcGIS, the study analyzes the spatiotemporal evolution characteristics of carbon and pollutant emissions from heavily polluting enterprises in the Yangtze River Economic Belt. This approach uncovers the internal “black box” through which digital transformation synergistically promotes pollution and carbon reduction, offering valuable insights for the comprehensive management of regional air pollution. Thirdly, the paper further investigates the factors intensifying the role of enterprise digital transformation in synergistic emission reduction and its underlying mechanisms. This deepens the understanding of the driving factors behind enterprise digital transformation and provides targeted policy recommendations.

## Theoretical mechanisms and hypotheses

### Theoretical mechanisms and hypotheses

Enterprise digital transformation refers to the deep integration of digital technologies into the entire production and operation process, driving fundamental changes in resource allocation methods, production organization, and governance structures through data empowerment, intelligent management, and business model innovation. In the context of high-pollution industries, digital transformation is primarily reflected in the extensive application of technologies such as artificial intelligence (AI), big data analytics, cloud computing, the Internet of Things (IoT), and automation control across production processes, management systems, and supply chains. At the macro level, the development of the digital economy effectively delivers environmental dividends, contributing to a reduction in the overall intensity of pollutant emissions and carbon emissions. At the micro level, enterprise digital transformation optimizes production processes through intelligent technologies and data-driven management, minimizes resource waste, and significantly reduces the marginal cost of managing pollutants and carbon emissions^50^. Moreover, there is a high overlap between the sources of air pollutants and greenhouse gas emissions, meaning that reducing pollutant emissions can simultaneously lower carbon dioxide emissions, thereby achieving synergistic effects^51^. Under a rational decision-making framework, enterprises with high marginal pollution control costs and subject to “dual constraints” often regard digital transformation as the optimal strategy for reducing compliance costs and addressing external environmental pressures^52^. Meanwhile, digital solutions enhance the precision of emission data collection and real-time monitoring, enabling enterprises to optimize carbon asset management and pollution control systems, further promoting the coordinated reduction of pollutant and carbon emissions^53^.

Specifically, the pathways through which enterprise digital transformation facilitates coordinated emission reduction are reflected in three key aspects: end-of-pipe treatment capacity enhancement, intelligent process control, and source prevention. First, regarding end-of-pipe treatment, digital transformation improves operational efficiency and financial performance, providing enterprises with more resources to introduce and upgrade end-treatment facilities such as high-efficiency desulfurization, denitrification equipment, and exhaust gas recovery systems, thereby strengthening pollution control capacity. Second, in terms of process control, IoT and AI-based control systems enable real-time monitoring and dynamic regulation of emissions data, ensuring pollutant discharge and energy consumption are maintained at optimal levels during production. Finally, in source prevention, digital technologies assist enterprises in optimizing production processes and management models by adopting cleaner production technologies and improving energy efficiency, thereby reducing the total amount of pollutant and carbon emissions at the source, lowering subsequent treatment costs, and achieving long-term, sustainable emission reduction goals. Based on this analysis, the following hypothesis is proposed:

#### Hypothesis 1

Digital transformation in enterprises facing dual constraints can effectively promote the synergistic reduction of both pollutants and carbon emissions.

Under the dual pressures of pollution control and carbon reduction, digital transformation is regarded as a crucial pathway for high-pollution enterprises to achieve both economic growth and environmental sustainability. Specifically, enterprise digital transformation facilitates the coordinated reduction of pollutant and carbon emissions through two primary channels: internal coordination and external coordination.

From the internal coordination perspective, digital transformation first optimizes resource allocation structures through the factor allocation effect. On one hand, enterprises increase investment in digital technologies and data resources, raising the proportion of data elements and knowledge-based technologies within production factors^54^. This restructuring of factor composition improves resource allocation efficiency and drives the internal optimization of enterprise resources^55,56^. Technologies such as big data analytics and simulation tools (e.g., digital twins) play a critical role during product design, enabling enterprises to adopt low-pollution raw materials and cleaner production processes, thereby reducing pollutants and carbon emissions at the source. Additionally, digitalized office procedures—such as paperless operations, remote work, and digital communications—help reduce paper consumption and the indirect carbon emissions associated with business travel, though their overall impact on high-pollution enterprises’ emissions remains relatively limited. By contrast, the digitalization of production processes plays a central role in achieving pollutant and carbon emission reductions. Through intelligent sensing, automated control systems, and AI-powered decision support, enterprises can monitor and dynamically adjust energy consumption and emissions in real-time, significantly improving production efficiency while reducing the intensity of emissions, including exhaust gases, wastewater, and CO₂. Recently, enterprise-level digital products driven by large models have emerged as a new trend in digital transformation. For example, new digital products represented by DeepSeek utilize historical data and environmental metrics to generate optimal production scheduling plans and emission control strategies, enabling enterprises to adopt low-carbon, efficient, and low-pollution production models. Furthermore, the extensive application of digital technologies supports enterprises in optimizing energy demand management, reducing dependence on high-energy, high-pollution production factors, and facilitating a shift from energy-intensive production modes to low-pollution, technology-intensive models. By comprehensively enhancing internal resource allocation and energy utilization efficiency, enterprises are able to lower environmental agency costs while simultaneously achieving coordinated governance of pollutant and carbon emissions^57^.

From the external coordination perspective, digital transformation significantly enhances the synergy between pollutant and carbon emission reduction through the effect of green technological innovation^58^. On one hand, digital transformation substantially improves enterprises’ research and development (R&D) capabilities and technological innovation capacity^59^, providing robust support for green technology innovation. By increasing R&D investment and optimizing technology development processes, enterprises are able to adopt more advanced green production technologies and environmentally friendly solutions, effectively reducing pollution intensity. On the other hand, the development and application of synergistic pollution-carbon reduction technologies often face high technical barriers and cost pressures when confined within a single enterprise. Digital transformation fosters collaborative innovation by creating technological platforms and information-sharing networks^60^, enabling the efficient flow of critical knowledge and information related to clean technology R&D and production across enterprises. This not only overcomes the isolation inherent in traditional production models, facilitating resource sharing and joint innovation, but also reduces the costs and risks associated with green technology innovation, accelerates technology diffusion, and improves the efficiency of collaborative governance. Additionally, digital transformation amplifies technology spillover effects^61,62^. Leveraging information technologies, enterprises can more effectively integrate external innovation resources, stay informed about market trends and technological advancements in real-time^63^, and promptly adjust their innovation strategies, promoting the large-scale application and diffusion of green technologies, thereby further enhancing the industry’s overall emission reduction synergy. Based on the above analysis, this paper proposes the following hypotheses.

#### Hypothesis 2

Enterprise digital transformation realizes pollution reduction and carbon reduction through two paths: enterprise internal synergy and external synergy.

Environmental regulation, as an external constraint and incentive mechanism, plays a pivotal moderating role in the relationship between enterprise digital transformation and the synergy of pollution and carbon reduction^64^.On the one hand, according to institutional theory, environmental regulation imposes stringent emission standards and “dual carbon” targets, placing constraints on enterprises’ pollutant emission behaviors^65^. This compels firms to optimize resource allocation and production processes to meet higher environmental performance requirements. On the other hand, moderate environmental regulation incentivizes enterprises to accelerate technological innovation and managerial upgrades^66^, fostering the deep integration of digital technologies into pollution control and carbon reduction efforts. Under regulatory pressure, enterprises increase their investments in digital resources and green technologies^67^, effectively reducing emission risks while achieving a synergy between production efficiency and environmental performance. Beyond institutional pressures, enterprises actively seek support from local governments to address resource constraints^68^, further enhancing the collaborative benefits of pollution and carbon reduction. Such initiatives also contribute to the achievement of enterprises’ sustainable development goals. Based on this analysis, the following hypothesis is proposed:

#### Hypothesis 3

Environmental regulation moderates the relationship between digital transformation and the synergistic effects of pollution and carbon reduction, promoting these synergistic improvements.

## Research design

### Benchmark model

The benchmark regression model for the effect of digital transformation on pollution reduction and carbon emission reduction in heavy-polluting enterprises is as follows. The benchmark model (1) is constructed to test hypothesis H1, In the model, “i” represents the enterprise, “t” represents the year, “Controls” represents all control variables, $\phi_ {\text{t}}$ representing time fixed effects, $\omega_{\text{s}}$ representing industry fixed effects, $\mu_ {\text{i}}$ representing individual fixed effects, and $\varepsilon_ {\text{it}}$ represents the random error term. The constant term is denoted by $\alpha_ {0}$, and the coefficients are denoted by α_1_, α_2_.

1 $$Synergy\;indexit = \alpha _{0} + \alpha _{1} EDT_{{it}} + \alpha _{2} Controls_{{it}} + \phi _{t} + \mu _{i} + \omega _{s} + \varepsilon _{{ist}}$$

### Data description

#### Dependent variables

(1) *Pollution emissions data*. This study, following the methodologies of Han^69^, further collected data by web scraping annual reports, sustainability reports, social responsibility reports, official websites of listed companies, and the public portal of the National Pollutant Discharge Permit Management Information Platform. Information related to environmental pollution from these reports and websites was used to supplement the pollution emission data. Based on the method proposed by Mao et.al^70^, this study categorizes corporate emissions into water pollution and air pollution, as shown in Table 1.

**Table 1:** The definition of synergy index.

Synergy index	Index	Weight
Air pollution	Sulfur dioxide emission intensity	14.023
	Nitrogen oxide emission intensity	14.007
	Particulate matter emission intensity	13.985
Water pollution	Chemical oxygen demand (COD) emission intensity	9.329
	Ammonia nitrogen emission intensity	14.007
	Total nitrogen emission intensity	14.764
	Total phosphorus emission intensity	14.662
Carbon pollution	Carbon dioxide emission intensity	5.193

(2) *Carbon emissions data*. At present, there is no dedicated database for compiling and collecting enterprise carbon emission data. Therefore, following Chapple’s methodology^71^, this study measures enterprise carbon emissions using carbon emission intensity. Drawing on the classification standards for carbon emission scopes from the studies of An and Wang^52,72^, this paper analyzes carbon emissions from combustion and fugitive emissions, waste emissions, and emissions from production processes. According to international standards, corporate carbon emissions can be categorized into three scopes: Scope 1 includes direct emissions from sources owned or controlled by the company; Scope 2 refers to indirect emissions resulting from the consumption of purchased heat and electricity; and Scope 3 covers other unspecified indirect emissions. Based on the “Corporate Greenhouse Gas Emission Accounting Methods and Reporting Guidelines” issued by the National Development and Reform Commission (NDRC) for various industries, this study separately calculates Scope 1 and Scope 2 emissions. The total carbon emissions are then obtained by summing the Scope 1 and Scope 2 emissions. According to the “IPCC Guidelines for National Greenhouse Gas Inventories”^73^, the carbon emissions calculation method for a fuel type is as follows:

2 $$E = AD \times EF$$

$AD$ represents the activity level data of consuming the fossil fuel, obtained by multiplying the fuel consumption by the average lower heating value. $EF$ represents the emission factor of the fossil fuel. The annual total carbon emissions of a company are calculated according to the formula, and its carbon emissions intensity is the ratio of the company’s carbon emissions to its annual operating costs.

(3) Synergy Index of corporate reduction of pollution and carbon emissions.The entropy method is used to calculate the weights of each variable and then calculate the overall index. The specific calculation process is as follows: First, the variables are dimensionless and then subjected to data standardization. The information entropy of the data($Ej$) is analyzed using Eq. (3).

3 $$E_{j} = - \frac{1}{{\ln n}}\sum\limits_{{i = 1}}^{n} {P_{{ij}} \ln (\frac{{x_{{ij}} }}{{\sum\limits_{{i = 1}}^{n} {x_{{ij}} } }})} ,i = 1,...n;j = 1,...m$$

$p_{ij}$ denotes the pollutant emission intensity. If $p_{ij} = 0$, then $\mathop {\lim }\limits_{pij \to 0} pij\ln p_{ij} = 0$ and calculate the utility value of information and compute the indicator weights by Eq. (4).

4 $$w_{j} = \frac{1 - E_{j}}{{\sum\limits_{j = 1}^{m} {(1 - Ej)} }},j = 1,...,m$$

5 $$S_{i} = \sum\limits_{{j = 1}}^{m} {w_{j} p_{{ij}} ,i = 1,...,n}$$

As greenhouse gas and environmental pollutant emissions are interconnected, caused by the same reasons, and share the same process^74^, the emissions of pollutants and carbon emissions from listed heavy polluting companies are measured by the entropy method. The three kinds of wastes (SO2, NOx, PM2.5) are the main components of air pollution and the major emissions of economic activities. In contrast, carbon emissions data, closely tied to corporate interests, are harder to obtain beyond government-mandated disclosures. Additionally, the accuracy of carbon emission calculations is limited, leading to lower overall information entropy and, thus, a lower weight in the indicator system. Table 1 outlines the weights assigned to the various indicators in the synergy index system. The synergistic pollution and carbon reduction index is calculated by a linear weighted summation based on Eq. (5).

### Explanatory variables

Although there is ongoing debate regarding the use of text analysis methods to measure digital transformation, the input–output method typically allocates industry-level digital investment indicators to the enterprise level based on relevant weights, rather than directly constructing metrics at the enterprise level^49^. This paper argues that text analysis methods provide a more precise reflection of an enterprise’s digitalization level. Drawing on the study by Wu et al.^75^, this research utilizes the China Digital Economy Research Database from the CSMAR database. Based on a feature-word map of “ABCD” technologies (Artificial Intelligence, Blockchain, Cloud Computing, and Big Data), the degree of enterprise digital transformation is measured across six dimensions. The detailed measurement framework is shown in Table 2.

**Table 2:** Definitions of enterprise digital transformation indicators.

Project	Content	Time
Strategic guidance	Management’s Digital Position Setup Table (annual), Management’s Digital Innovation Frequency Statistics Table (annual), Management’s Digital Innovation Indicator Details Table (annual), Management’s Digital Innovation Orientation Indicator Table (annual)	2010–2022
Technology driven	Technology Empowerment Indicator Details Table (annual), Artificial Intelligence Technology Frequency Statistics Table (annual), Blockchain Technology Frequency Statistics Table (annual), Cloud Computing Technology Frequency Statistics Table (annual), Big Data Technology Frequency Statistics Table (annual) | 2010–2022 Organizational Empowerment | Digital Capital Investment Plan Statistics Table (annual), Digital Human Resource Investment Plan Statistics Table (monthly), Digital Infrastructure Construction Situation Table (annual), Digital Infrastructure Construction Indicator Details Table (annual), Technology Innovation Base Construction Situation Table (annual)	2010–2022
Organizational empowerment	Digital Capital Investment Plan Statistics Table (annual), Digital Human Resource Investment Plan Statistics Table (monthly), Digital Infrastructure Construction Situation Table (annual), Digital Infrastructure Construction Indicator Details Table (annual), Technology Innovation Base Construction Situation Table (annual)	2010–2022
Environmental support	Invention Patent Application Statistics Table by Industry (annual), R&D Activity Situation Table by Industry (annual), New Product Development and Sales Situation Table by Industry (annual), Digital Technology Intensity Statistics Table by Industry (annual), Digital Capital Investment Intensity Statistics Table by Industry (annual), Human Capital Investment Intensity Statistics Table by Industry (annual), Optical Cable Line Length Statistics Table by City (annual), Mobile Switch Capacity Statistics Table by City (annual), Internet Broadband Access User Scale Statistics Table by City (annual), Mobile Internet User Scale Statistics Table by City (annual)	2010–2022
Digital achievements	Digital Innovation Standard Work Statistics Table (annual), Digital Innovation Paper Quantity Statistics Table (annual), Digital Invention Patent Authorization Situation Table (annual), Digital Innovation Qualification Statistics Table (annual), National Digital Awards Statistics Table (annual)	2010–2022
Digital applications	Digital Application Indicator Details Table (annual), Technology Innovation Frequency Statistics Table (annual), Process Innovation Frequency Statistics Table (annual), Business Innovation Frequency Statistics Table (annual)	2010–2022
Digital transformation index	Strategic Guidance Score Table (annual), Technology Driven Score Table (annual), Organizational Empowerment Score Table (annual), Environmental Support Score Table (annual), Digital Achievements Score Table (annual), Digital Applications Score Table (annual), Enterprise Digital Transformation Index Statistics Table (annual)	2011–2022

### Mediation variables

According to existing research^76^, data on enterprise joint technological innovation is sourced from the CNRDS database. Green agency cost is calculated as the ratio of environmental management costs (e.g., greening and sanitation fees) to total operating income:

GAc = environmental management costs / total operating income. A lower value indicates better integration of green innovation resources within the enterprise.

### Moderator variable

The National Development and Reform Commission (NDRC) launched the Low-Carbon City Pilot Program in 2010 to support China’s carbon peak commitment. Successive batches of pilot regions were introduced in 2012 and 2017 to encourage green technological innovation, improve low-carbon systems, and achieve energy conservation and emission reduction. In June 2013, the State Council issued the Air Pollution Prevention and Control Action Plan to establish coordinated pollution control in the Beijing-Tianjin-Hebei and Yangtze River Delta regions. Cities along the Yangtze River Economic Belt were given specific targets to reduce key pollutants, excellent particulate matter (PM2.5), and inhalable particulate matter (PM10).

To capture the synergistic effects between “pollution reduction” and “carbon reduction” policies, this paper incorporates two moderating variables—air pollution control policies and the low-carbon city pilot policy—under environmental regulations. For publicly listed companies in regions where these policies were implemented, the environmental policy dummy variable is set to 1, and before policy approval, it is set to 0.

### Control variable

This study conducts empirical research from a micro perspective. Based on previous research^46^, it selects enterprise size, debt-to-assets ratio, return on assets, cash flow ratio, revenue growth rate, and shareholding ratio of the largest shareholder as control variables. The explanations for each control variable are detailed in Table 3.

**Table 3:** Descriptive statistic.

Variables	Variable description	N	Mean	Median	Min	Max	Std. Dev
Synergy index	Synergy index of pollution reduction and carbon emission Reduction.entropy method for constructing an index system	4831	0.993	0.996	0.933	0.999	0.009
EDT	Digital transformation index system	4831	0.304	0.284	0.234	0.477	0.058
Co-innovation	Green technology patents jointly applied for by listed heavy-polluting enterprises are represented in the form of logarithms plus one	4831	0.192	0.060	0.000	2.699	0.453
Green agency	GAc = environmental management costs / total operating income	4831	0.010	0.010	0.000	2.507	0.010
Size	Enterprise Scale. Represented by the Natural logarithm of total assets at the end of the period	4831	22.193	22.025	20.143	25.459	1.164
Lev	Debt-to-asset ratio.represented by the ratio of total liabilities to total assets	4831	0.388	0.371	0.060	0.868	0.191
Roa	Return on assets. represented by the ratio of net profit to total assets	4831	0.051	0.047	− 0.147	0.219	0.061
Cashflow	The ratio of the net cash flow from operating activities of the enterprise to total assets	4831	0.063	0.060	− 0.115	0.240	0.064
Growth	Operating income growth rate, represented by the ratio of the increase in operating income this year to the total operating income of the last year	4831	0.154	0.100	− 0.385	1.824	0.319
Indep	Proportion of independent directors, represented by the ratio of independent directors to the total number of board members	4831	37.072	33.330	33.330	50.000	4.718
Top1	Proportion of shares held by the largest shareholder, represented by the ratio of the number of shares held by the largest shareholder to the total number of shares	4831	33.796	32.206	9.203	73.132	13.868

### Data source

This study focuses on A-share listed companies in the Yangtze River Economic Belt that face “dual constraints.” These industries are classified based on the “Industry Classification and Management Catalogue for Environmental Protection Check of Listed Companies,” published by the Ministry of Ecology and Environment in June 2008, and include 16 categories, such as thermal power, steel, and cement. To identify heavily polluting enterprises facing “dual constraints,” industries with specific production characteristics, such as steel, chemical, and power, were selected as the research sample.

The sample period of this study is from 2011 to 2022. The research data is derived from the China Stock Market & Accounting Research Database (CSMAR), and interpolation methods fill in some missing data. The descriptive statistics of the relevant variables are shown in the Table 3. The sample of this study consists of panel data of A-share listed companies in heavy-polluting industries. The primary financial data of listed companies mainly come from CSMAR, and the pollution emission data of listed companies are primarily obtained from annual reports, corporate social responsibility reports, and corporate websites. Before conducting the econometric analysis, this study removed samples that underwent special treatment from the financial industry, ST, and PT, as well as samples with severe missing variables, and the sample was also subject to 1% level truncation.

### Spatiotemporal analysis

#### The spatiotemporal evolution of enterprises’pollution and carbon reduction synergy

Air pollution is highly mobile and widespread, with solid regional links between heavy-polluting companies. Investigating the spatiotemporal evolution of pollution and carbon emission reductions in the Yangtze River Economic Belt offers valuable insights into the spatial distribution and regional transfer of point-source pollution. The indicator calculations analyze the spatiotemporal evolution of synergistic effects among these companies through inverse distance weighting interpolation. Figure 1 illustrates a spatial clustering pattern, where heavily polluting enterprises are more concentrated in the east and sparser in the west of the Yangtze River Economic Belt.

**Fig. 2:**
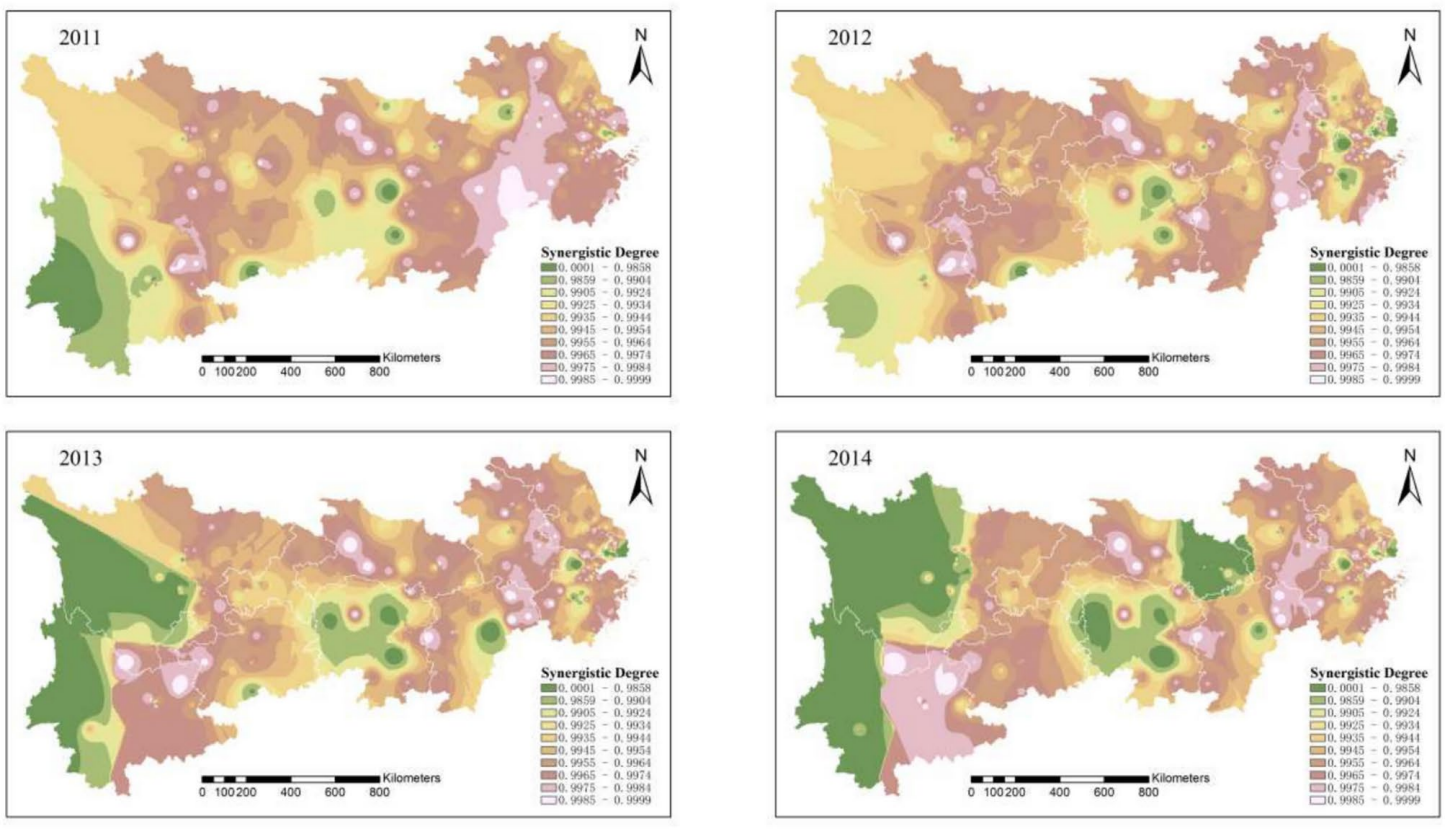
Synergistic degree of pollution reduction and carbon reduction in heavily polluting enterprises in the Yangtze River Economic Belt from 2011 to 2014. Software version: ArcMap 10.8, URL: https://www.esri.com/zh-cn/arcgis/products/index This map is produced based on the standard map with the review number GS(2024)0650, downloaded from the National Geographic Information Public Service Platform (website: https://www.tianditu.gov.cn/), and the base map has not been modified.

Figure 2 indicates that from 2011 to 2012, China’s development model was relatively extensive, and the middle and lower reaches of the Yangtze River did not exhibit significant synergistic effects. The downstream Yangtze River Delta region experiences severe environmental pollution, where pollution sources spread out in a blob-like pattern, and the level of coordinated pollution and carbon reduction control is low. However, small areas of low pollution began to emerge in 2013. This change can be attributed to the government’s issuance of the “Twelfth Five-Year Plan for Controlling Greenhouse Gas Emissions” at the end of 2012, which aimed to accelerate the transformation of the economic development model. The plan imposed strict CO2 emission reduction targets, strengthening environmental supervision and penalties for enterprises.

**Fig. 3:**
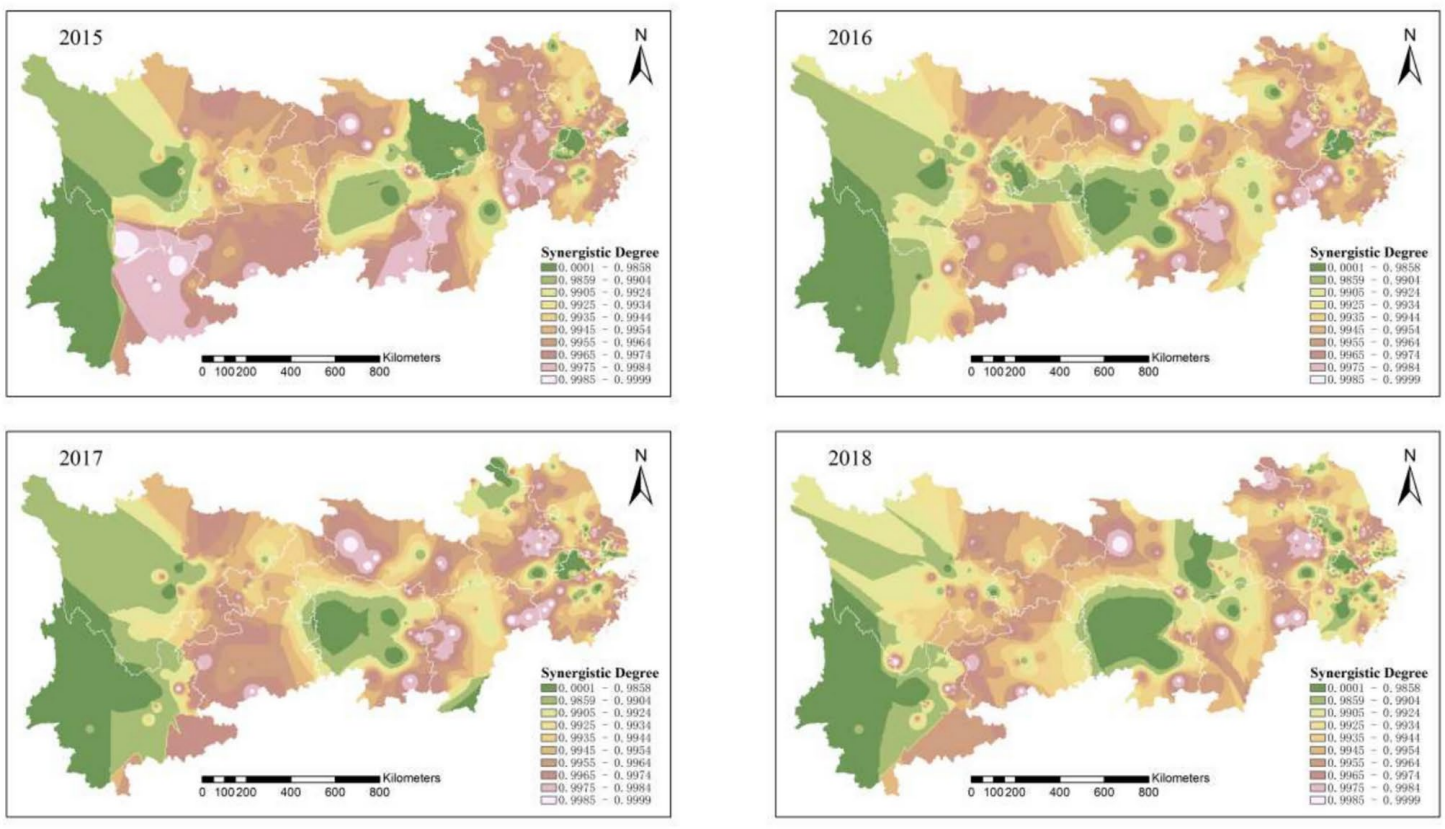
Synergistic degree of pollution reduction and carbon reduction in heavily polluting enterprises in the Yangtze River Economic Belt from 2015 to 2018. Software version: ArcMap 10.8, URL: https://www.esri.com/zh-cn/arcgis/products/index This map is produced based on the standard map with the review number GS(2024)0650, downloaded from the National Geographic Information Public Service Platform (website: https://www.tianditu.gov.cn/), and the base map has not been modified.

Figure 3 illustrates the synergy between pollution and carbon emission reductions in the Yangtze River Economic Belt from 2015 to 2018. Large areas of low synergy and high pollution are steadily shrinking. In the upper Yangtze River, emissions from heavily polluting enterprises are now under control, showing a solid synergy between pollution and carbon reduction. In the middle and lower reaches, pollution levels decrease annually, except for leading enterprise clusters. Some heavily polluting enterprises have relocated, contributing to an overall improvement in the region’s synergistic governance of pollution and carbon reduction.

**Fig. 4:**
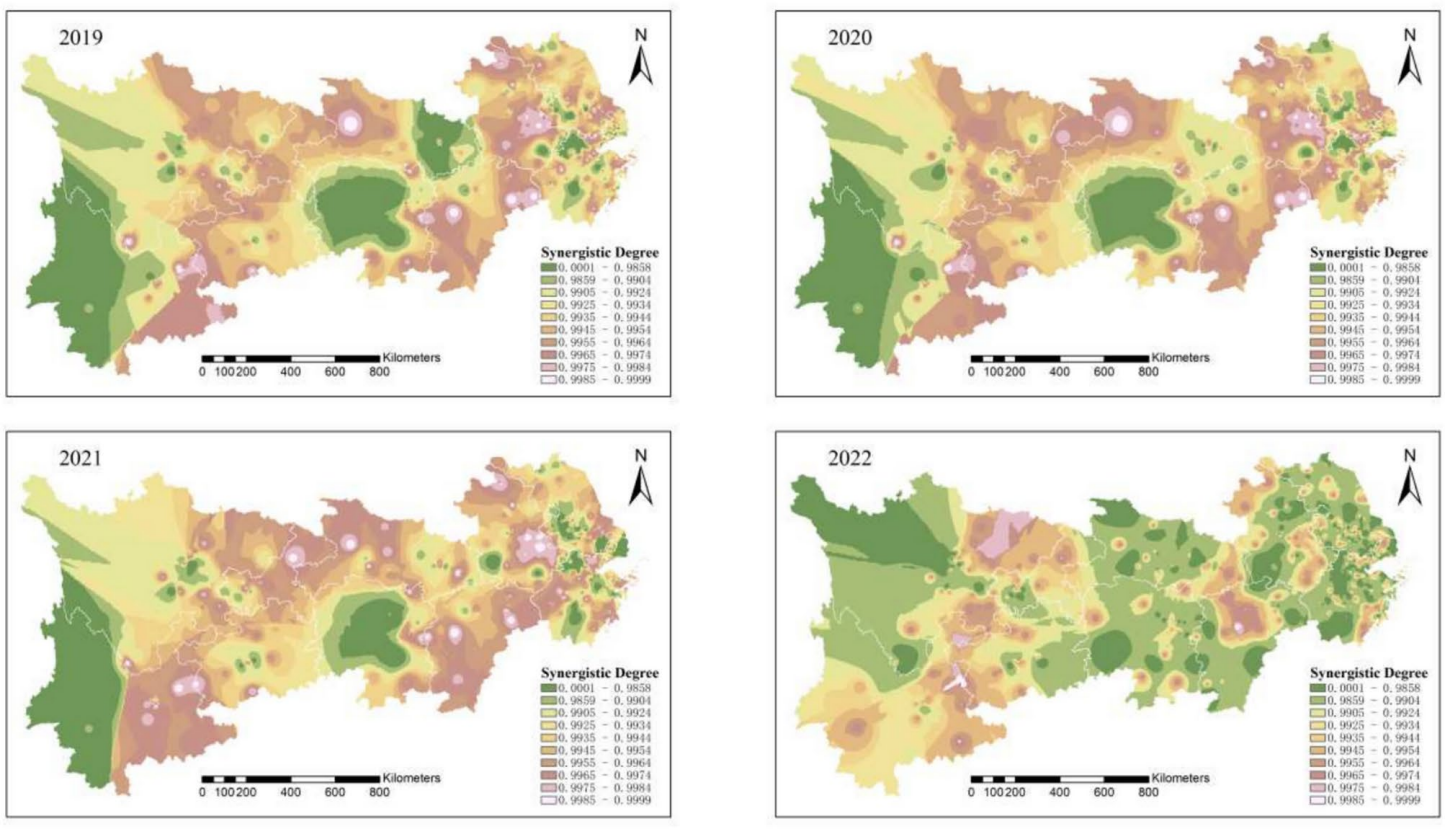
Synergistic degree of pollution reduction and carbon reduction in heavily polluting enterprises in the Yangtze River Economic Belt from 2019 to 2022. Software version: ArcMap 10.8, URL: https://www.esri.com/zh-cn/arcgis/products/index This map is produced based on the standard map with the review number GS(2024)0650, downloaded from the National Geographic Information Public Service Platform (website: https://www.tianditu.gov.cn/), and the base map has not been modified.

Between 2019 and 2022, pollution emissions in the study area sharply declined. As shown in Fig. 4, by 2022, the synergy between pollution reduction and carbon reduction among heavily polluting enterprises in the Yangtze River Economic Belt had significantly improved, with areas of low synergy being sparsely and sporadically distributed.

A comprehensive analysis shows that the pollution and carbon reduction synergy index in the Yangtze River Economic Belt follows a spatial gradient, with a “gradual decrease from upstream to downstream.” Over time, the pattern shifted from “large contiguous areas of low synergy” to “small, dispersed areas of high synergy.” By 2022, the region displayed a “concentrated low pollution” characteristic, indicating a strong synergy between pollution and carbon reduction. Overall, from 2011 to 2022, the spatial extent and intensity of pollution and carbon emissions from heavily polluting enterprises in the Yangtze River Economic Belt have significantly declined.

### Spatiotemporal evolution of digital transformation in heavily polluting enterprises

**Fig. 5:**
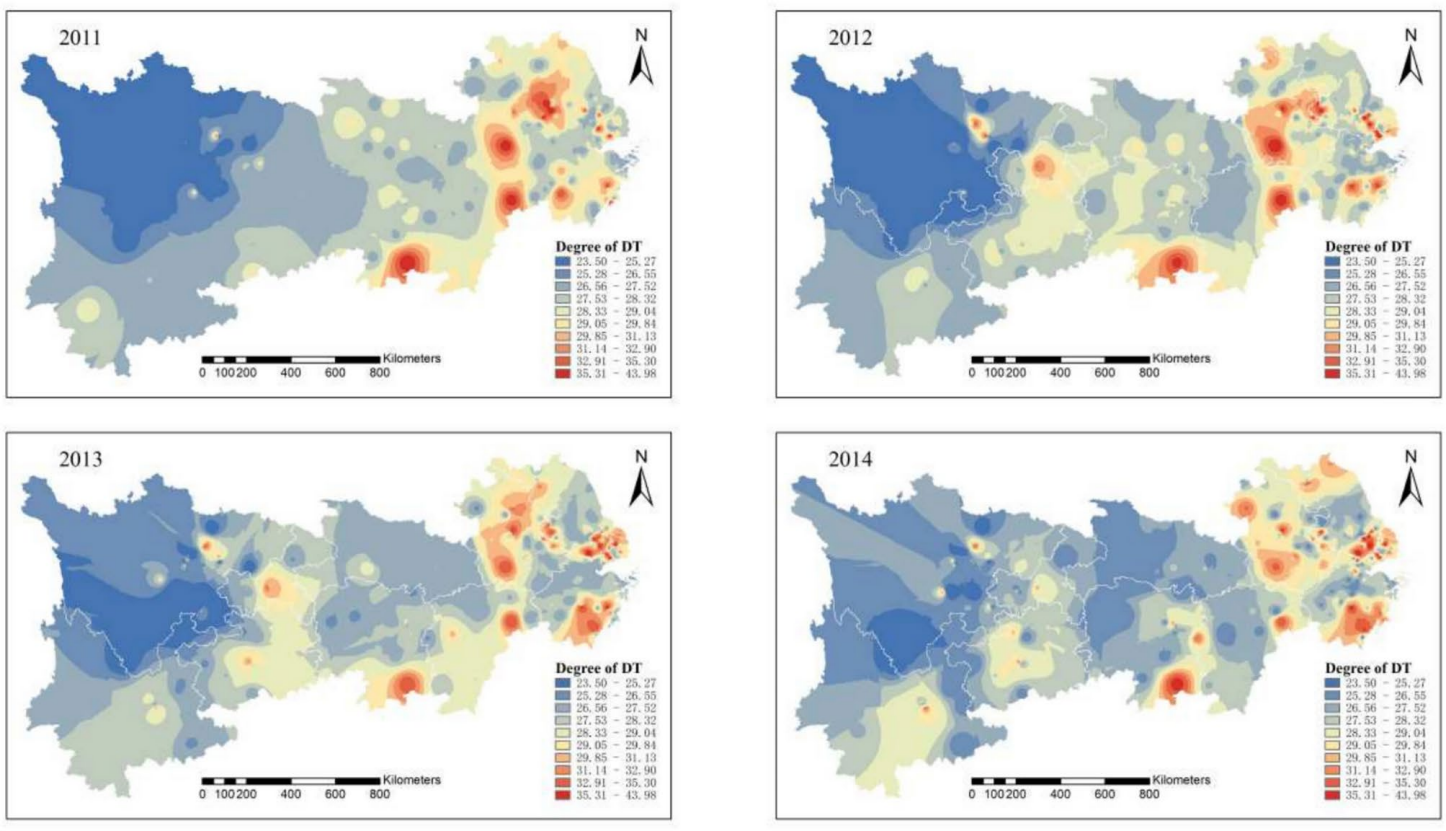
Degree of Digital Transformation in Heavily Polluting Enterprises in the Yangtze River Economic Belt from 2011 to 2014. Software version: ArcMap 10.8, URL: https://www.esri.com/zh-cn/arcgis/products/index This map is produced based on the standard map with the review number GS(2024)0650, downloaded from the National Geographic Information Public Service Platform (website: https://www.tianditu.gov.cn/), and the base map has not been modified.

This study uses the inverse distance weighting method to visualize the level of digitalization. It provides an intuitive understanding of the regional digitalization characteristics among these companies and explores the potential for synergistic effects on pollution and carbon reduction. As shown in Fig. 5, from 2011 to 2014, digitalization in the middle and upper reaches of the Yangtze River was still in its early stages. Most digital transformation efforts were concentrated in the lower reaches, particularly in regions surrounding Shanghai, where digitalization levels were higher.

**Fig. 6:**
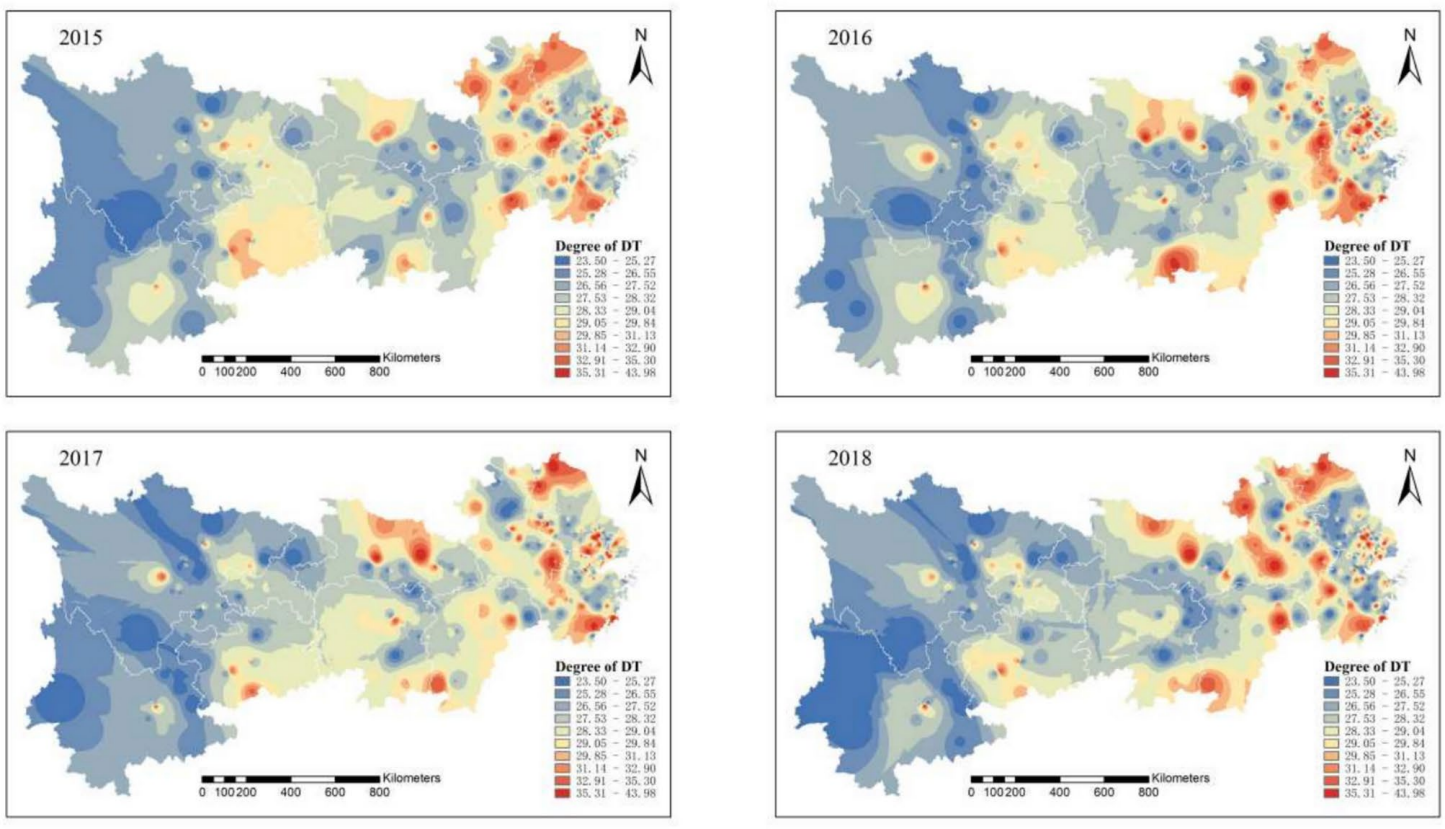
Degree of Digital Transformation in Heavily Polluting Enterprises in the Yangtze River Economic Belt from 2015 to 2018. Software version: ArcMap 10.8, URL: https://www.esri.com/zh-cn/arcgis/products/index This map is produced based on the standard map with the review number GS(2024)0650, downloaded from the National Geographic Information Public Service Platform (website: https://www.tianditu.gov.cn/), and the base map has not been modified.

Analysis of Fig. 6 reveals that from 2015 to 2018, the degree of digitalization among enterprises radiated from the lower reaches of the Yangtze River towards the middle and upper reaches, forming a “T”-shaped distribution of highly digitalized enterprises. The digitalization process in the middle reaches of the Yangtze River significantly increased during this period. In the upstream regions, particularly Yunnan Province, the average digitalization level of enterprises in other areas remained relatively low.

**Fig. 7:**
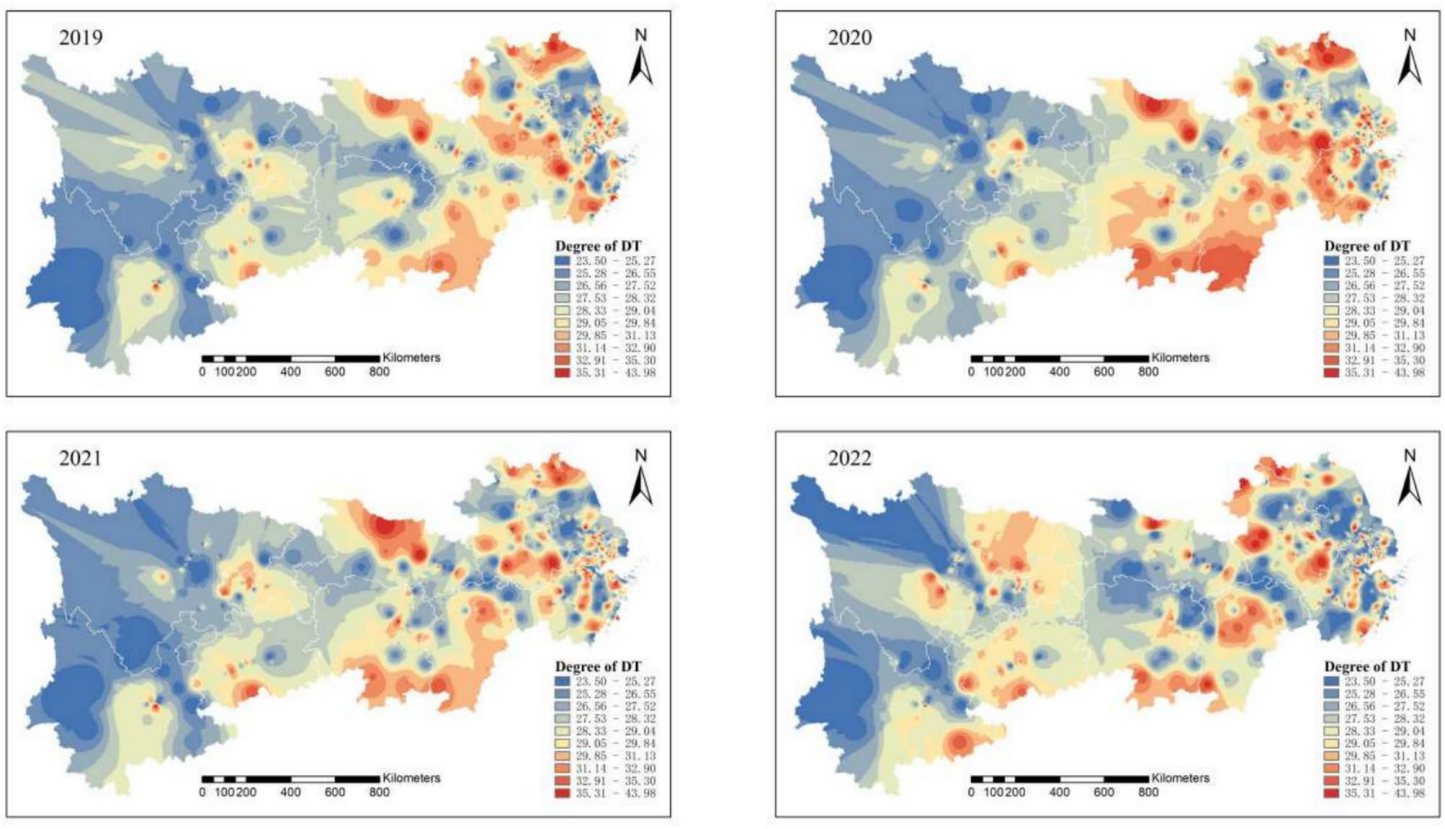
Degree of Digital Transformation in Heavily Polluting Enterprises in the Yangtze River Economic Belt from 2019 to 2022. Software version: ArcMap 10.8, URL: https://www.esri.com/zh-cn/arcgis/products/index This map is produced based on the standard map with the review number GS(2024)0650, downloaded from the National Geographic Information Public Service Platform (website: https://www.tianditu.gov.cn/), and the base map has not been modified.

Figure 7 shows that digitalization among heavily polluting enterprises in the lower reaches of the Yangtze River is expanding outward in a drip-like pattern. This growth is likely driven by the “trickle-down effect” and learning from clustered enterprises, which accelerates digitalization around high-tech core firms. Environmental regulations have also prompted some companies in Shanghai and surrounding areas to relocate, shifting the focus of digitalization towards the middle and upper reaches. In the middle reaches, highly digitalized enterprises are distributed in sheet-like patterns.

Overall, there is a strong correlation between the digital transformation of heavily polluting enterprises and the synergy of pollution and carbon reduction in the Yangtze River Economic Belt. Digitalization has significantly supported efforts to reduce both pollution and carbon emissions, promoting synergy and improving regional efficiency in these areas.

## Results and discussion

### Baseline regression

Table 4 presents the results of the baseline regression. This conclusion aligns with the perspective of Liu et al.^77^, who argue that digital transformation (DT) can mitigate the conflict between the dual goals of pollution and carbon reduction and promote synergistic pollution and carbon reduction (SPCR) Enterprise digitalization significantly enhances firms’ ability to acquire, integrate, and utilize external data and information resources for technological innovation. It also improves the precision of matching production with market demand, thereby increasing economies of scale and factor allocation efficiency in production processes. These advancements contribute to reducing pollution and improving the level of synergistic emission reduction. By optimizing resource utilization and operational efficiency, enterprise digitalization markedly slows the growth rate of pollutant emissions, thereby facilitating the achievement of synergistic pollution and carbon reduction goals. Based on column (1), control variables are gradually added until all control variables are included, and the coefficient of EDT becomes − 0.008, significant at the 1% level. This indicates that enterprise digital transformation hurts pollution emissions and promotes the synergy reduction of pollution and carbon emissions. Hypothesis H1 is supported.

**Table 4:** Descriptive statistics.

Variables	(1)	(2)	(3)	(4)	(5)	(6)	(7)	(8)	(9)
	Synergy index	Synergy index	Synergy index	Synergy index	Synergy index	Synergy index	Synergy index	Synergy index	Synergy index
Digital Transformation(EDT)	− 0.007***	− 0.008***	− 0.008***	− 0.008***	− 0.008***	− 0.008***	− 0.008***	− 0.008***	− 0.008***
	(− 2.813)	(− 3.490)	(− 3.509)	(− 3.502)	(− 3.563)	(− 3.494)	(− 3.488)	(− 3.687)	(− 3.687)
Size		0.003***	0.003***	0.002***	0.003***	0.002***	0.002***	0.002***	0.002***
		(7.648)	(7.896)	(7.585)	(7.644)	(7.208)	(7.269)	(7.359)	(7.359)
Lev			0.002	0.003*	0.003*	0.002	0.002	0.002	0.002
			(1.242)	(1.802)	(1.767)	(1.338)	(1.363)	(1.355)	(1.355)
Roa				0.006**	0.004*	0.000	0.000	0.000	0.000
				(2.262)	(1.785)	(0.174)	(0.171)	(0.181)	(0.181)
Clashflow					0.004**	0.004***	0.004***	0.004***	0.004***
					(2.409)	(2.720)	(2.722)	(2.676)	(2.676)
Growth						0.002***	0.002***	0.002***	0.002***
						(3.513)	(3.512)	(3.564)	(3.564)
Indep							− 0.000	− 0.000	− 0.000
							(− 0.785)	(− 0.796)	(− 0.796)
Top1								− 0.000*	− 0.000*
								(− 1.829)	(− 1.829)
Constant	0.996***	0.934***	0.936***	0.939***	0.938***	0.943***	0.944***	0.944***	0.944***
	(1351.474)	(115.113)	(125.250)	(128.328)	(127.149)	(129.808)	(133.995)	(135.770)	(135.770)
Year FE	Yes	Yes	Yes	Yes	Yes	Yes	Yes	Yes	Yes
Industry FE	Yes	Yes	Yes	Yes	Yes	Yes	Yes	Yes	Yes
ID FE	Yes	Yes	Yes	Yes	Yes	Yes	Yes	Yes	Yes
Observations	4831	4831	4831	4831	4831	4831	4831	4831	4831
R-squared	0.824	0.838	0.839	0.839	0.840	0.842	0.842	0.842	0.842

Robust standard errors in parentheses; ***p < 0.01, **p < 0.05, *p < 0.1

### Robustness tests

#### Replacement of explained variables

In the baseline regression, the dependent variable was substituted to ensure robustness. First, following the study by Mao et al.^70^, enterprise pollution emissions are categorized into chemical oxygen demand (COD) and ammonia nitrogen emissions from industrial wastewater, as well as sulfur dioxide (SO_2_) and nitrogen oxides (NO_x_) from industrial exhaust. Drawing on the methodology from prior research^78^, pollutant emissions were standardized using pollutant equivalent values as defined in the "Regulations on the Collection and Use of Pollution Discharge Fees." When this composite pollution indicator was reintroduced into the regression model, the results remained robust. Second, SO_2_, being the primary air pollutant, and CO_2_ emission data were used to replace the synergy index for robustness testing. Both variables were found to be statistically significant. The robustness test results validate Hypothesis 1 and further confirm the synergistic relationship between air pollutants and CO_2_ emissions. During the process of digital transformation, enterprises not only reduce air pollutant emissions but also achieve a simultaneous decrease in CO₂ emissions. This indicates that digital transformation in enterprises facing “dual constraints” has a synergistic effect on pollution reduction and emission control.

### Replacement of explanatory variables

Referring to the study by Yu et al.^79^, this paper adopts the entropy method to evaluate the process of enterprise digital transformation (EDT) by measuring digital transformation input–output. First, drawing on the research by Tao et al.^80^, the ratio of intangible assets related to digital transformation to total intangible assets of listed companies is used as an input indicator for measuring enterprise digital transformation. Additionally, following the methodology proposed by Zhou^81^, patent data from the China National Intellectual Property Administration (CNIPA) is utilized. The main classification codes of patents are matched with the “Statistical Classification of the Digital Economy and its Core Industries 2021” to obtain data on digital economy-related patents of listed companies. This serves as the output indicator for measuring enterprise digital transformation. Table 5 presents the weights of the input and output indicators in the measurement framework. When the newly constructed digital transformation indicators are incorporated into the regression model, the results remain robust, confirming the validity of the conclusions. Table 6 provides the results of the robustness test.

**Table 5:** Digital transformation weighting ratio.

Digital transformation weighting ratio (WDigital)				
Indicator selection	Calculation method	Type	Sign	Weight (%)
Digital intangible assets	Proportion of intangible assets related to digitalization to total intangible assets in listed companies	Input	Positive	39.46
Digital economy patents	Number of digital economy-related patent applications by listed companies	Output	Positive	60.54

**Table 6:** Robustness Test.

Variables	(1)	(2)	(3)	(4)
	Leap	SO_2_	CO_2_	Synergy index
EDT	− 0.001**	− 0.380***	− 0.525**	
	(− 2.524)	(− 3.895)	(− 2.339)	
WDigital				− 0.007***
				(− 3.014)
Constant	0.146***	6.520***	− 4.701***	0.944***
	(119.106)	(31.502)	(-6.268)	(129.187)
Control variables	Yes	Yes	Yes	Yes
Year FE	Yes	Yes	Yes	Yes
Industry FE	Yes	Yes	Yes	Yes
ID FE	Yes	Yes	Yes	Yes
Observations	4831	4831	4831	4831
R-squared	0.938	0.617	0.910	0.841

Robust standard errors in parentheses; ***p < 0.01, **p < 0.05, *p < 0.1

### Endogeneity test

In order to address the possible endogenous problem, the study constructs instrumental variables for digital transformation. By controlling for enterprise, industry, and year-fixed effects and using robust standard errors at the enterprise level, the model is estimated using two-stage least squares (2SLS).

Based on the research of Han et al.^68^, this study selects the variables of terrain slope, which represents the natural geographical features of the city where the company is located, to construct the instrumental variable for enterprise digital transformation. City terrain slope, as a natural geographical condition that reflects regional terrain complexity to some extent, has a certain impact on the installation and debugging of digital infrastructure, potentially increasing the difficulty and cost of infrastructure construction. However, it does not directly affect a company’s pollution emissions. Therefore, this instrumental variable can satisfy the requirements of relevance and homogeneity. To avoid automatically excluding instrumental variables that do not vary over time in fixed effects estimation, this study constructs an instrumental variable denoted as Slope&IPR with both spatial and temporal dimensions by multiplying city terrain slope (Slope) with the national internet penetration rate (IPR) at the national level over the years.

According to Table 7, the interaction term between city Slope and the IPR over the years shows a positive correlation with the EDT and has an F-value of 67.229, meeting the weak instrument variable test and satisfying the validity test conditions. After controlling the endogeneity issues, the impact coefficient of digital transformation on the reduction of pollution and carbon emissions in heavy-polluting companies is significantly negative. This indicates that digital transformation has a significant negative effect on carbon emissions and pollution emissions in these companies, which is consistent with the baseline regression results.

**Table 7:** Endogeneity test.

	(1)	(2)
	EDT	Synergy index
Slope&IPR	0.001***	
	(3.861)	
EDT		− 0.047**
		(− 2.229)
Constant	0.175***	
	(3.780)	
Control variables	Yes	Yes
Year FE	Yes	Yes
Industry FE	Yes	Yes
ID FE	Yes	Yes
Observations	4831	4831
R-squared	0.698	− 0.023

Robust standard errors in parentheses; ***p < 0.01, **p < 0.05, *p < 0.1

### Analysis of influencing mechanisms

This section follows the recommendations of Jiang et al. on mediation effect analysis^82^. Mediator variables that directly and significantly influence enterprise pollution reduction and can measure the effects of green technological innovation and factor allocation are selected as dependent variables. These variables are used to examine the mechanisms through which enterprise digitalization and enterprise relational networks impact pollution reduction.

Green innovative technologies play a critical role in enhancing production efficiency and facilitating the upgrade to low-carbon green technologies. As previously discussed, enterprises that have completed capital accumulation are not subject to the “dual constraints” of emissions reduction. However, due to the difficulty of overcoming technical barriers and the lack of substantial capital, less resource-rich enterprises often engage in external collaborative green technology innovation to reduce the costs of technological innovation and achieve synergistic emission reduction. Therefore, joint green technology innovation among enterprises is used as a mediating variable for testing. Within enterprises, the factor allocation effects of digitalization improve resource utilization efficiency, gradually reducing the need for outsourced green services. To examine this effect, the integration of internal resources is tested using the cost of green agency as a mediating variable. The results in Table 8 confirm a significant mediating effect. This indicates that digital transformation promotes synergistic pollution and carbon reduction through internal and external collaboration, supporting Hypothesis H2.

**Table 8:** Test of influencing mechanisms.

	Test of influencing mechanisms	
	Co-innovation	Green agency
EDT	0.457**	− 0.005***
	(2.446)	(− 2.877)
Constant	− 1.334***	− 0.024***
	(− 2.810)	(− 4.800)
Control variables	Yes	Yes
Year FE	Yes	Yes
Industry FE	Yes	Yes
ID FE	Yes	Yes
Observations	4831	4831
R-squared	0.544	0.709

Robust standard errors in parentheses; ***p < 0.01, **p < 0.05, *p < 0.1

### Moderation effect test

Table 9 reports the moderating effects, in conjunction with the previous analysis, that environmental regulatory policies may influence the impact of digital transformation on synergistic pollution and carbon reduction. According to the moderation effect results in Table 9, the interaction terms between digital transformation and the low-carbon city pilot policy and critical air pollution control regions are negatively correlated at the 5% significance level. The possible reason is that environmental regulation policies restrict enterprise emissions at the source. Given the synergistic effect between pollution reduction and carbon reduction, both “pollution reduction” policies and “carbon reduction” policies play a role in promoting coordinated emission reductions. This suggests that firms located within low-carbon city pilot areas and critical air pollution control regions are more likely to leverage digital transformation to enhance the synergistic effects of pollution and carbon reduction, thereby supporting Hypothesis H3.

**Table 9:** Test of moderation effect.

	Moderation effect test	
	Synergy index	Synergy index
EDT	− 0.008***	− 0.010***
	(− 3.569)	(− 6.069)
Air pollution plan	− 0.003***	
	(− 4.318)	
EDT × air pollution plan	0.006**	
	(2.488)	
Low-carbon		− 0.004***
		(− 3.429)
EDT × low-carbon		0.007**
		(2.090)
Constant	0.949***	0.948***
	(141.391)	(141.240)
Control variables	Yes	Yes
Year FE	Yes	Yes
Industry FE	Yes	Yes
ID FE	Yes	Yes
Observations	4831	4831
R-squared	0.845	0.846

Robust standard errors in parentheses; ***p < 0.01, **p < 0.05, *p < 0.1

### Further analysis

#### External heterogeneity analysis

This study differentiates between the lower, middle, and upper reaches of the Yangtze River Economic Belt to explore regional differences in the impact of digital transformation on pollution and carbon reduction among heavily polluting enterprises. The regression results in Table 10 show that the inhibitory effect of digital transformation on emissions is more pronounced in the middle and lower reaches. In contrast, the effect is not significant in the upper reaches.

**Table 10:** Test heterogeneity test.

	Upstream	Midstream	Downstream	Large Size	Small Size	Weak financing constraints	Strong financing constraints
	Synergy index	Synergy index	Synergy index	Synergy index	Synergy index	Synergy index	Synergy index
EDT	− 0.002	− 0.017**	− 0.008***	− 0.011**	− 0.008***	− 0.008**	− 0.008***
	(− 0.424)	(− 2.199)	(− 3.384)	(− 2.118)	(− 10.666)	(− 2.001)	(− 11.094)
Constant	0.960***	0.935***	0.936***	0.873***	0.982***	0.886***	0.974***
	(69.572)	(46.220)	(109.265)	(43.971)	(259.809)	(56.198)	(168.414)
Control variables	Yes	Yes	Yes	Yes	Yes	Yes	Yes
Year FE	Yes	Yes	Yes	Yes	Yes	Yes	Yes
Industry FE	Yes	Yes	Yes	Yes	Yes	Yes	Yes
ID FE	Yes	Yes	Yes	Yes	Yes	Yes	Yes
Observations	927	764	3140	2371	2403	2478	2258
R-squared	0.837	0.842	0.846	0.856	0.784	0.859	0.878

Robust standard errors in parentheses; ***p < 0.01, **p < 0.05, *p < 0.1

From an analytical perspective, the middle and lower regions boast more developed economies and higher levels of digitalization. The lower reaches, in particular, serve as a central hub for digital technology research and innovation, with well-established digital infrastructure, abundant data resources, and a high degree of enterprise concentration, fostering closer collaboration among firms. Furthermore, the sample size of enterprises in the lower reaches accounts for approximately two-thirds of the total, strengthening the digital transformation capabilities of heavily polluting enterprises in this region in transitioning to low-carbon, low-pollution operations.

### Internal heterogeneity analysis

Table 10 shows that large-scale enterprises have a more substantial capacity to adapt to environmental regulations due to their economies of scale. These firms already have robust compliance systems and more resources to handle environmental penalties or carbon trading costs, reducing their incentive to rely on digital transformation for pollution and carbon reduction. Instead, they rely on existing pollution control measures to meet compliance. Additionally, many large-scale enterprises have already achieved low levels of pollutant emissions and carbon footprints through previous adoption of environmental technologies and efficient management. As a result, the marginal benefits of further digital transformation are limited. This explains why digital transformation is relatively weaker in promoting pollution and carbon reduction for large-scale enterprises.

Similarly, firms facing more substantial financing constraints urgently need to improve production efficiency and resource utilization to reduce costs. As a result, they are more proactive in adopting digital tools to optimize resource allocation and meet pollution and carbon reduction goals. In contrast, firms with weaker financing constraints tend to rely on traditional capital investments to fulfill environmental requirements. Moreover, firms with solid financing constraints face tremendous pressure from environmental regulations but need more resources for substantial investments to meet emission reduction targets quickly. For these companies, digital transformation becomes the primary way to enhance resource efficiency and reduce pollution control costs.

## Conclusions and implications

### Discussion

#### Research comparison

Through empirical testing, our findings demonstrate that enterprise digital transformation significantly promotes the synergy of pollution and carbon reduction. These results are consistent with existing literature. Hu et al. constructed a multi-period difference-in-differences model to examine the mechanisms and impacts of digitalization on the synergy of pollution and carbon reduction^38^. They introduced technological innovation and energy efficiency as mediating variables to explore the role of digitalization in achieving synergistic outcomes. Similarly, Chen et al., based on panel data from 108 Chinese cities between 2006 and 2019, utilized a coupling coordination degree model to assess the city-level synergy of pollution and carbon reduction^83^. They further employed a Geodetector model to identify driving factors such as the digital economy and technological innovation.

While many scholars prefer using provincial- or municipal-level data to study the interplay between digitalization and pollution-carbon reduction synergy, the primary driving force behind digitalization lies at the enterprise level. Enterprises play a central role in adopting new technologies, innovating processes, and transforming market strategies. Therefore, this study utilizes micro-level enterprise data, providing a more comprehensive analysis of the impact of digitalization on coordinated pollution and carbon emission reductions compared to the aforementioned studies.

### Limitations and prospects

This study has certain limitations. The sample is limited to A-share listed heavily polluting enterprises in China, excluding general industrial enterprises across China and other regions globally. Additionally, the use of text analysis to measure enterprise digital transformation is subject to debate, indicating that the methods for evaluating digital transformation require further refinement.

Future research could address these gaps by expanding the sample to include all listed companies and small- and medium-sized enterprises (SMEs), incorporating international comparisons, and considering additional forms of pollutants. Such efforts would help to bridge existing research gaps and enrich the theoretical and empirical understanding of the synergy between pollution and carbon reduction from a more comprehensive perspective.

### Conclusions

1. Using the entropy method, we constructed a synergy index for pollution and carbon reduction. Spatiotemporal analysis revealed that heavily polluting enterprises in the Yangtze River Economic Belt exhibit a spatial pattern characterized by "dense in the east and sparse in the west," with pollution sources spreading outward in clusters.
2. To further explore the underlying theoretical mechanisms through which enterprise digitalization influences synergistic pollution and carbon reduction, this study introduced joint technological innovation and green outsourcing costs as mechanism variables. The results indicate that under the dual pressures of “pollution reduction” and "carbon reduction," enterprise digitalization primarily operates through external collaborative technological innovation and internal resource integration, thereby further reducing carbon and pollutant emissions.
3. Additionally, the study finds that air pollution prevention policies and low-carbon city pilot programs play a critical role in accelerating the impact of enterprise digitalization on synergistic emission reduction, promoting greater reductions. Regarding regional heterogeneity, the middle and lower reaches of the Yangtze River demonstrate more pronounced synergistic effects of pollution and carbon reduction. At the enterprise level, smaller firms with strong financing constraints achieve more significant synergistic efficiency in pollution and carbon reduction through digitalization.

This study deepens the understanding of the intricate relationships among enterprise digitalization, technological innovation, environmental regulation policies, and the synergy of pollution and carbon reduction.

### Policy recommendations

Based on the above research findings, the following countermeasures and recommendations are proposed.

First, capitalizing on the synergy between pollution reduction and carbon mitigation, governments can achieve dual emission reduction outcomes by enforcing only one type of environmental regulation, either targeting “pollution reduction” or “carbon mitigation.” Furthermore, in regions with dense concentrations of heavily polluting enterprises, governments can establish benchmark companies that excel in implementing coordinated pollution reduction and carbon mitigation strategies. The successful practices of these benchmark companies should be systematically summarized and broadly disseminated. By adopting this "point-to-line-to-surface" approach, more enterprises can be guided to achieve coordinated emission reductions.

Second, optimize the Support Environment for Digital Transformation. Governments should enhance their support for the research and development of digital technologies in heavily polluting industries, thereby encouraging traditional enterprises to accelerate their digital transformation. Digital technologies offer the potential to significantly improve emission reduction capabilities. Specific actions include establishing collaborative platforms to foster joint technological innovation among enterprises, promoting the widespread sharing and adoption of green production technologies, and ultimately driving both emission reductions and operational efficiency improvements.

Third, tailor Support for Small and Micro-Enterprises and Address Regional Differences. For small-scale, financially constrained high-pollution enterprises, governments should strengthen policy support and provide substantial financial and technological assistance to ensure their successful digital transformation, thereby achieving notable synergistic emission reduction benefits. For example, in the middle and lower reaches of the Yangtze River Economic Belt, stricter oversight of pollutant and carbon emissions should be implemented. Conversely, in the upper reaches, the emphasis should be placed on accelerating the digital transformation of heavily polluting enterprises to maximize the effectiveness of coordinated pollution and carbon emission reductions. These measures aim to achieve a dual-win outcome, balancing economic development with environmental sustainability.

## Data Availability

The data that support the findings of this study are available from the corresponding author upon reasonable request.
